# Cumulative Excess Body Mass Index and MGUS Progression to Myeloma

**DOI:** 10.1001/jamanetworkopen.2024.58585

**Published:** 2025-02-07

**Authors:** Lawrence Liu, Nikhil Grandhi, Mei Wang, Ekaterina Proskuriakova, Theodore Thomas, Martin W. Schoen, Kristen M. Sanfilippo, Kenneth R. Carson, Alissa Visram, Celine Vachon, Graham Colditz, Murali Janakiram, Mengmeng Ji, Su-Hsin Chang

**Affiliations:** 1 Research Service, St Louis Veterans Affairs Medical Center, St Louis, Missouri; 2 City of Hope Comprehensive Cancer Center, Duarte, California; 3 Department of Medicine, Washington University School of Medicine, St Louis, Missouri; 4 Division of Public Health Sciences, Department of Surgery, Washington University School of Medicine, St Louis, Missouri; 5 Department of Medicine, Mount Sinai Hospital, Chicago, Illinois; 6 Department of Medicine, St Louis University School of Medicine, St Louis, Missouri; 7 Division of Hematology and Oncology, Northwestern University Feinberg School of Medicine, Chicago, Illinois; 8 Division of Hematology, University of Ottawa, Ottawa Hospital Research Institute, Ottawa, Ontario, Canada; 9 Division of Epidemiology, Department of Quantitative Sciences, Mayo Clinic, Rochester, Minnesota

## Abstract

**Importance:**

Obesity is a risk factor associated with multiple myeloma (MM) and its precursor, monoclonal gammopathy of unknown significance (MGUS). However, it is unclear how cumulative exposure to obesity affects the risk of MGUS progression to MM.

**Objective:**

To determine the association of cumulative exposure to excess body mass index (EBMI), defined as BMI (calculated as weight in kilograms divided by height in meters squared) greater than 25, with risk of MGUS progression to MM.

**Design, Setting, and Participants:**

This cohort study included patients with MGUS, including immunoglobin G, immunoglobin A, or light chain MGUS, from the nationwide US Veterans Health Administration database from October 1, 1999, to December 31, 2021. A published natural language processing–assisted model was used to confirm diagnoses of MGUS and progression to MM. Data were analyzed from February 12 to November 4, 2024.

**Exposures:**

Cumulative EBMI was calculated by area under the curve of measured BMI subtracting the reference BMI at 25 during the first 3 years after MGUS diagnosis.

**Main Outcomes and Measures:**

The main outcome was progression from MGUS to MM. Multivariable Fine-Gray time-to-competing-event analyses, with death as the competing event, were used to determine associations.

**Results:**

The cohort included 22 429 patients with MGUS (median [IQR] age, 70.5 [63.5-77.9] years; 21 613 [96.4%] male), with 8329 Black patients (37.1%) and 14 100 White patients (62.9%). There were 4862 patients (21.7%) with reference range BMI (18.5 to <25), 7619 patients (34.0%) with BMI 25 to less than 30, and 8513 patients (38.0%) with BMI 30 or greater at the time of MGUS diagnosis. Compared with reference range BMI at MGUS diagnosis, patients with BMI 25 to less than 30 (adjusted hazard ratio [aHR], 1.17; 95% CI, 1.03-1.34) or 30 or greater (aHR, 1.27; 95% CI, 1.09-1.47) at MGUS diagnosis had higher risk of progression to MM. In patients with reference range BMI at MGUS diagnosis, each 1-unit increase of EBMI per year was associated with a 21% increase in progression risk (aHR, 1.21; 95% CI, 1.04-1.40). However, for patients with BMI 25 or greater at MGUS diagnosis, the incremental risk associated with cumulative EBMI exposure was not statistically significant.

**Conclusions and Relevance:**

This cohort study found that, for patients with BMI 18.5 to less than 25 at the time of MGUS diagnosis, cumulative exposure to BMI 25 or greater was associated with an increased risk of progression. These findings suggest that for these patients, maintaining a healthy and stable weight following MGUS diagnosis may prevent progression to MM.

## Key Points

**Question:**

To what extent is cumulative exposure to excess body mass index (BMI, calculated as weight in kilograms divided by height in meters squared) associated with risk of progression from monoclonal gammopathy of undetermined significance (MGUS) to multiple myeloma (MM)?

**Findings:**

In this cohort study of 22 429 patients with MGUS, among those with BMI of 18.5 to less than 25 at MGUS diagnosis, each 1-unit increase of excess BMI (ie, >25) per year was associated with a statistically significant 21% higher risk of progression to MM.

**Meaning:**

These findings suggest that maintaining a healthy and stable weight may prevent progression of MGUS to MM.

## Teaser

This cohort study examines the association of cumulative exposure to excess body mass index with the risk of progression of monoclonal gammopathy of unknown significance (MGUS) to multiple myeloma.

## Introduction

Multiple myeloma (MM) is a common hematologic neoplasm.^1^ Although the survival rates have improved,^2,3^ the management for MM still incurs the highest costs of care among all cancers, with a recent estimate of $757 386 per patient, with relapsed or refractory MM at a follow-up of 22 months.^4^ MM is preceded by an asymptomatic condition known as *monoclonal gammopathy of undetermined significance* (MGUS).^5,6,7,8,9^ The prevalence of MGUS is approximately 3% in the US population older than 50 years.^10^ Approximately 20% of patients with MGUS progress to MM or related diseases in their lifetime.^11^ As such, studying MGUS progression is critical for finding prevention strategies. Identifying risk factors for progression can guide the frequency of monitoring to facilitate timely diagnosis of MM and inform prevention strategies.

Obesity is identified as one of the few modifiable risk factors associated with MM by the International Agency for Research on Cancer with strong evidence,^12^ and studies have reported on the positive association between obesity at a single time point and the progression of MGUS to or development of MM.^13,14,15^ Several biochemical studies have demonstrated that this could be related to circulating adipokines, immunoparesis, and other pathways.^16,17,18,19,20,21,22^ However, to our knowledge, no study has evaluated to what extent the duration or magnitude of obesity is associated with progression. One population-based study by Marinac et al^23^ assessed the association between self-reported body shape trajectory and MM risk, and findings indicated that lower, stable weight may be beneficial in the prevention of MM.^23^ However, Marinac et al^23^ did not assess MGUS to MM progression.

The goal of our study is to assess the association between cumulative exposure of excess body mass index (EBMI) and MGUS to MM progression. The findings of this study are critical for a better understanding of how cumulative exposure of EBMI may change progression risk among patients with MGUS and inform public health interventions focused on prevention.

## Methods

This cohort study was approved by the Washington University School of Medicine institutional review board and St Louis VHA’s Research and Development Committee and institutional review board, which also waived the requirement for informed consent because data were deidentified. Data were reported according to the Strengthening the Reporting of Observational Studies in Epidemiology (STROBE) reporting guideline.

### Data, Study Population, and Design

We conducted a cohort study using data from the nationwide US Veterans Health Administration (VHA), including all 21 Veterans Integrated Service Networks. The VHA Corporate Data Warehouse (CDW) database was launched as a centralized storage of diagnostic data in 1999. As such, serum protein electrophoresis, immunofixation, and other diagnostic data are only available from 1999 and after.^24,25^ This study included patients with confirmed MGUS diagnosis from October 1, 1999, to December 31, 2021, in the CDW database (Figure). Diagnoses of MGUS were identified and confirmed by a published classification model using natural language processing (NLP) and machine learning along with diagnosis date, MGUS subtype, and monoclonal protein (M-protein) concentration at diagnosis.^26,27^ We included patients with immunoglobulin (Ig) G, IgA or light chain MGUS, since IgM MGUS typically progresses to lymphoma and rarely to MM and IgD and IgE MGUS are very rare.^28,29^ Biclonal MGUS was not included since the monoclonal protein value can be reported unreliably. We only included Black or White patients since the proportions of other races in the VHA population are low. Race and ethnicity information was self-reported as recorded in the electronic health records in the VHA and pulled from the CDW database, including the following categories: American Indian or Alaska Native, Asian, Black or African American, declined, Native Hawaiian or Other Pacific Islander, unknown, White, and White not of Hispanic origin. The unknown and declined groups were listed as missing. American Indian or Alaska Native, Asian, and Native Hawaiian or Other Pacific Islander were grouped as other race and ethnicity. Race was recoded based on a published algorithm and categorized into Black and White.^30^ Furthermore, we used the following exclusion criteria: patients with no BMI measurements within the observation window (ie, 3 years following MGUS diagnosis); patients who progressed within 6 months of MGUS diagnosis, as these patients may have already progressed to MM at baseline; and patients with MM treatment before MGUS diagnosis, as these patients likely had already progressed.

**Figure.**
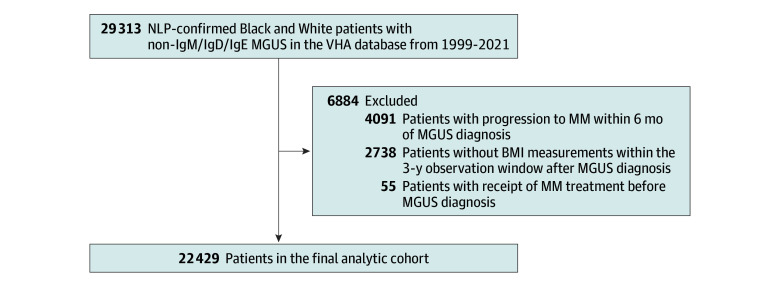
Flowchart of Patient Accrual for Patients Diagnosed With Monoclonal Gammopathy of Undetermined Significance (MGUS) in the Veterans Health Administration (VHA) Database, 1999-2021 BMI indicates body mass index; Ig, immunoglobin; MM, multiple myeloma; NLP, natural language processing.

### Exposure

The exposure variable was cumulative exposure of EBMI over 3 years following MGUS diagnosis. Objectively measured height and weight were collected from CDW domains in the VHA. BMI was calculated as weight in kilograms divided by height in meters squared. EBMI was defined as measured BMI subtracting the reference BMI at 25^2,31^; only positive values contributed to EBMI, and negative values were recorded as 0, ie, EBMI_t_ = max(measured BMI_t_ − 25, 0), where *_t_* is any time point within the observation window where BMI measurements are available. To capture the cumulative exposure of EBMI, we calculated the area under the curve (AUC) of EBMI trajectory during the 3-year observation window after MGUS diagnosis. The EBMI-years captures the degree and duration of excess BMI over 3 years following MGUS diagnosis. For example, an adult with a BMI of 30 for 3 years would accumulate 15 EBMI-years ([30 − 25] EBMI × 3 years). For individuals who progressed to MM or censored within 3 years, the last measured BMI was extrapolated to the entire 3-year observation window to ensure everyone had a full 3-year window for AUC and reduce confounding resulting from a smaller AUC for patients with a follow-up less than 3 years. Details of the calculations are provided in the eFigure in Supplement 1.

### Outcome

The primary outcome was time from MGUS diagnosis to progression to MM, if any. Patients were followed-up from MGUS diagnosis to MM, death, or censoring, whichever occurred first. The date of last data retrieval was February 12, 2024. The 3-year observation window of EBMI is independent from the follow-up time. For MM diagnosis, 2 criteria were required: (1) patients had at least 1 *International Classification of Diseases, Ninth Revision (ICD-9)* code of 203.0 or *International Statistical Classification of Diseases and Related Health Problems, Tenth Revision (ICD-10)* code of C90.0 for MM and (2) the receipt of any MM-specific treatment listed in eTable 1 in Supplement 1. The identified diagnoses were then confirmed by a NLP-assisted machine learning–based classification model along with diagnosis date.^26,27,32^ Our NLP model was able to confirm MM diagnoses with high performance (recall of 100% and precision of 92.3%).^32^

### Covariates

We included the following covariates in the multivariable analysis: age at MGUS diagnosis, sex (male, female), race (Black, White), MGUS type (IgA, IgG, light chain), baseline BMI, M-protein level at MGUS diagnosis (<1.5 g/dL, ≥1.5 g/dL), presence of diabetes, presence of anemia, presence of chronic kidney disease (CKD), and Charlson Comorbidity Index. Baseline BMI was calculated using the height that was most frequently recorded and weight measurements taken within 1 year before or after MGUS diagnosis and closest to the time of MGUS diagnosis. Baseline BMI was then categorized as less than 18.5, 18.5 to less than 25 (reference group), 25 to less than 30, and 30 or greater. Baseline Charlson Comorbidity Index was calculated with comorbidities present within 1 year prior to MGUS diagnosis,^33,34^ as well as the presence of diabetes and CKD.

### Statistical Analysis

Individual characteristics of the study sample were summarized using descriptive statistics. A multivariable competing risk Fine-Gray analysis was performed to estimate the association between cumulative EBMI exposure and progression with death as the competing event,^35,36^ adjusting for known covariates associated with progression. Missing values for categorical variables were categorized as unknown status to preserve the sample size.

Variance inflation factor (VIF) was calculated to measure the degree of collinearity variables. A VIF greater than 5 indicates high correlation of the variables.^37^ Point-Biserial correlation coefficients were also calculated, with a coefficient of greater than 0.7 considered indicative of collinearity.^38^ For cumulative EBMI exposure and baseline BMI, VIF was less than 5, indicating no evidence for collinearity; consequently, the AUCs of EBMI and baseline BMI were analyzed in the competing risk model simultaneously. Furthermore, the interaction between baseline BMI and cumulative EBMI exposure was tested. Subgroup analyses stratified by baseline BMI were performed if they were determined to improve the model explanatory power (based on log-likelihood values).

All statistical tests were 2-sided. Statistical significance was determined using α = .05. All statistical analyses were performed using SAS software version 9.2 (SAS Institute). Data were analyzed from February 12 to November 4, 2024.

## Results

There were 29 313 patients with a diagnosis of MGUS in the VHA between 1999 and 2021 whose MGUS subtype was not IgM, IgD, or IgE. We excluded a total of 6884 patients to ensure data quality and relevance: 2738 patients without BMI measurements within the 3-year observation window after MGUS diagnosis; 4091 patients with progression within 6 months of MGUS diagnosis, as these patients may have already progressed to MM; and 55 patients with MM treatment data before MGUS diagnosis, as progression likely had occurred. Our final analytic cohort included 22 429 patients with MGUS (median [IQR] age, 70.5 [63.5-77.9] years; 21 613 [96.4%] male) (Figure and Table 1).

**Table 1.** Baseline Characteristics of the Analytic Cohort of Patients in the Veterans Health Administration Diagnosed With MGUS, 1999-2021

Characteristic	Patients, No. (%) (N = 22 429)
BMI at MGUS diagnosis	
<18.5	357 (1.6)
18.5 to <25	4862 (21.7)
25 to <30	7619 (34.0)
≥30	8513 (38.0)
Unknown	1078 (4.8)
Monoclonal protein level at MGUS diagnosis, g/dL	
<1.5	14 799 (66.0)
≥1.5	1480 (6.6)
Unknown	6150 (27.4)
Sex	
Female	816 (3.6)
Male	21 613 (96.4)
MGUS Type	
IgG	17 601 (78.5)
IgA	2897 (12.9)
Light chain	1931 (8.6)
Race	
Black	8329 (37.1)
White	14 100 (62.9)
Age at MGUS diagnosis, y	
Median (IQR)	70.5 (63.5 to 77.9)
≤65	6651 (29.7)
>65	15 778 (70.4)
Diabetes^a^	
Yes	11 396 (50.8)
No	11 033 (49.2)
Anemia^a^	
Yes	6849 (30.5)
No	15 580 (69.5)
Chronic kidney disease^a^	
Yes	6117 (27.3)
No	16 312 (72.7)
Progression	
Censored	9194 (41.0)
Confirmed progression	1845 (8.2)
Death without progression	11 390 (50.8)
EBMI AUC (1 EBMI-year), median (IQR)	0.7 (0.0-3.3)
Charlson Comorbidity Index, median (IQR)	2.0 (0.0-4.0)
Follow-up time, median (IQR), y	5.4 (3.2-8.5)
Time to Progression, median (IQR), y	2.7 (1.2-5.4)

Abbreviations: AUC, area under the curve; BMI, body mass index (calculated as weight in kilograms divided by height in meters squared); Ig, immunoglobin; MGUS, monoclonal gammopathy of undetermined significance.

^a^ The presence of diabetes, anemia, and chronic kidney disease was identified with the disease present within 1 year prior to MGUS diagnosis.

The cohort included 8329 Black patients (37.1%) and 14 100 White patients (62.9%). Among the cohort, 4862 patients (21.7%) had BMI 18.5 to less than 25 (reference group), 7619 patients (34.0%) had BMI 25 to less than 30 and 8513 patients (38.0%) had BMI 30 or greater at the time of MGUS diagnosis. Most patients had an M-protein level less than 1.5 g/dL (14 799 patients [66.0%]) and MGUS types were predominantly IgG (17 601 patients [78.5%]), followed by IgA (2897 patients [12.9%]) and light chain (1931 patients [8.6%]). Many patients had diabetes (11 396 patients [50.8%]), anemia (6849 patients [30.5%]), or CKD (6117 patients [27.3%]) at time of MGUS diagnosis. Median (IQR) follow-up was 5.4 (3.2-8.5) years. Progression was observed in 1845 patients (8.2%), with a median (IQR) time to progression of 2.7 (1.2-5.4) years.

Both VIF values (all <5) and correlation coefficient (0.36) indicated no evidence for collinearity between AUC of EBMI and baseline BMI. Therefore, both baseline BMI and cumulative exposure were included in the multivariable analysis. The multivariable analysis results show that the incremental risk attributed to cumulative EBMI was not statistically significant (multivariable-adjusted hazard ratio [aHR], 0.99; 95% CI, 0.98-1.00;) after adjusting for baseline BMI at MGUS diagnosis. Having BMI 25 to less than 30 at MGUS diagnosis was associated with a 17% increase in progression risk (aHR, 1.17; 95% CI, 1.03-1.34) (Table 2). Having BMI 30 or greater at the diagnosis of MGUS was associated with a 27% increase in progression risk (aHR, 1.27; 95% CI, 1.09-1.47) (Table 2). The inclusion of the interaction between baseline BMI and AUC of EBMI in the model significantly improved its explanatory power, suggesting an interplay between baseline BMI and subsequent BMI changes in the progression of MGUS to MM (eTable 2 in Supplement 1).

**Table 2.** Multivariable Analysis of the Risk of Progression to MM in Patients Diagnosed With MGUS in the Veterans Health Administration, 1999-2021

Variable	aHR (95% CI)	*P* value
Cumulative excess BMI exposure (per 1 EBMI-year)^a^	0.99 (0.98-1.00)	.08
BMI at MGUS diagnosis		
<18.5	0.87 (0.56-1.35)	.53
18.5 to <25	1 [Reference]	
25 to <30	1.17 (1.03-1.34)	.02
≥30	1.27 (1.09-1.47)	.002
M-protein level at MGUS diagnosis, g/dL		
<1.5	1 [Reference]	
≥1.5	5.51 (4.90-6.19)	<.001
Sex		
Female	1 [Reference]	
Male	1.57 (1.21-2.05)	<.001
MGUS type		
Ig G	1 [Reference]	
Ig A	1.46 (1.29-1.65)	<.001
Light chain	0.95 (0.79-1.14)	.59
Race		
White	1 [Reference]	
Black	1.15 (1.04-1.26)	.005
Age at MGUS diagnosis, per 1-y	0.98 (0.97-0.98)	<.001
Charlson Comorbidity Index	0.83 (0.80-0.86)	<.001
Diabetes	1.18 (1.08-1.30)	<.001
Anemia	1.13 (1.02-1.26)	.02
Chronic kidney disease	1.13 (0.98-1.30)	.10

Abbreviations: aHR, multivariable-adjusted hazard ratio; AUC, area under the curve; BMI, body mass index (calculated as weight in kilograms divided by height in meters squared); EBMI, excess body mass index; Ig, immunoglobin; MGUS, monoclonal gammopathy of undetermined significance; MM, multiple myeloma.

^a^ The cumulative EBMI exposure was calculated by AUC of all available measured BMI within the 3 years following MGUS diagnosis subtracted by 25 to generate a trajectory.

Stratification by baseline BMI found that for patients with MGUS with BMI 18.5 to less than 25, each 1-unit increase of EBMI-years was associated with a 21% increase in progression risk (aHR, 1.21; 95% CI, 1.04-1.40) (Table 3). In patients with MGUS with BMI 25 or greater at baseline, the incremental risk attributed to cumulative EBMI, after accounting for baseline BMI, was not statistically significant (aHR, 0.99; 95% CI: 0.98-1.00).

**Table 3.** Multivariable Subgroup Analysis of the Risk of Progression to MM Among Patients in the Veterans Health Administration Diagnosed With MGUS Stratified by Baseline BMI Status, 1999-2021

Variable	Normal BMI at baseline (n = 4862)		Overweight/obese at baseline (n = 16 132)	
	aHR (95% CI)	*P* value	aHR (95% CI)	*P* value
Cumulative EBMI exposure, per 1 EBMI-year^a^	1.21 (1.04-1.40)	.01	0.99 (0.98-1.00)	.11
M-protein level at MGUS diagnosis, g/dL				
<1.5	1 [Reference]	<.001	1 [Reference]	<.001
≥1.5	5.03 (3.88-6.51)		5.49 (4.79-6.30)	
Sex				
Female	1 [Reference]	.047	1 [Reference]	.006
Male	2.10 (1.01-4.35)		1.54 (1.14-2.08)	
MGUS type				
Ig G	1 [Reference]	NA	1 [Reference]	NA
Ig A	1.49 (1.13-1.97)	.005	1.44 (1.24-1.66)	<.001
Light chain	1.02 (0.68-1.55)	.92	0.96 (0.78-1.18)	.68
Race				
Black	1.26 (1.02-1.57)	.04	1.14 (1.02-1.28)	.02
White	1 [Reference]		1 [Reference]	
Age at MGUS diagnosis, per 1-y	0.98 (0.97-0.98)	<.001	0.98 (0.97-0.98)	<.001
Charlson Comorbidity Index	0.72 (0.65-0.81)	<.001	0.85 (0.82-0.88)	<.001
Diabetes	1.25 (0.99-1.57)	.06	1.15 (1.03-1.28)	.01
Anemia	1.17 (0.93-1.48)	.17	1.10 (0.97-1.25)	.13
Chronic kidney disease	1.02 (0.69-1.52)	.92	1.11 (0.95-1.30)	.20

Abbreviations: aHR, multivariable-adjusted hazard ratio; AUC, area under the curve; EBMI, excess body mass index; Ig, immunoglobin; MGUS, monoclonal gammopathy of undetermined significance; MM, multiple myeloma.

^a^ The EBMI AUC was calculated by obtaining every BMI data point (calculated as weight in kilograms divided by height in meters squared) within the 3-year observation window following MGUS diagnosis and subtracting by 25 to generate a trajectory.

## Discussion

This cohort study is the first study to our knowledge to quantify the association between cumulative exposure to BMI 25 or greater and the progression from MGUS to MM. Patients with MGUS and baseline BMI 25 or greater had a 17% to 27% higher risk of progression to MM compared with patients with baseline BMI within the reference range. Progression risk attributed to cumulative EBMI, after accounting for baseline BMI, was negligible. However, in patients with baseline BMI within the reference range (22% of patients with MGUS), each increase of EBMI-year following MGUS diagnosis was associated with a 21% increase in the risk of progression. Our study quantified the increased risk of progression of MGUS to MM associated with both the duration and magnitude of the exposure of elevated BMI, instead of analyzing a binary classification of BMI at a single time point. The findings suggest that both the duration and magnitude of BMI elevation are modifiable risk factors associated with the progression to MM.

Obesity remains one of the few known modifiable risk factors associated with MGUS progression. To our knowledge, only a few studies have reported a positive association between high BMI and risk of MGUS progression.^13,14,39^ These studies provided crucial insight on the increased risk associated with excess BMI (BMI >25) measured at the time of MGUS diagnosis. However, previous studies did not include the measure of cumulative excess BMI and thus the risk associated with excess BMI was integrated into the exposure at 1 time point—usually time of MGUS diagnosis or time of survey. Our study further quantified the association of dose-dependent exposure of EBMI over time with progression risk. This suggests that the detrimental association of EBMI is cumulative, rather than at 1 time point.

Our findings inform management at the time of MGUS diagnosis and may facilitate more effective intervention in these patients. Low-risk MGUS is currently managed with follow-up at 6 months and then every 2 to 3 years if stable, while yearly follow-up is recommended for patients classified as having intermediate- or high-risk MGUS based on International Myeloma Working Group guidelines.^40^ Our study indicates that during the progression from asymptomatic MGUS to symptomatic MM, frequent ambulatory weight monitoring may be an affordable and beneficial intervention, especially for patients with BMI within the reference range at MGUS diagnosis. This strategy empowers patients to actively participate in their health management and provides continuous data that could be critical in monitoring. Patients with MGUS may also benefit from diet and lifestyle counseling and programs aimed at achieving a healthy and stable weight.^41,42^

For patients who were already overweight or obese at MGUS diagnosis, it would be of interest to investigate their cumulative exposure to elevated BMI prior to the MGUS diagnosis. This study did not focus on this aspect because our primary goal was to study prevention strategies for patients at the time of MGUS diagnosis. Nonetheless, by understanding how prediagnosis and postdiagnosis BMI management affects MGUS incidence and progression to MM, we could inform more effective interventions that could potentially delay or prevent the onset of MM in this patient population. Additionally, the findings of this study only reflect the association between excess BMI and progression; BMI reduction was not studied. Future studies focusing on whether weight loss or maintaining a stable weight can delay or prevent the biological transition toward MM are needed.

The strengths of our study include the analysis of the magnitude and duration of exposure, measured by the cumulative exposure of EBMI, which contributes to the literature evaluating BMI and MM development.^23^ Our method allowed for quantification of cumulative exposure of EBMI to evaluate its association with MGUS progression. Another strength was the use of a patient cohort from nationwide data, thus ensuring sufficient statistical power with a large sample size. Furthermore, the use of a validated NLP-assisted machine learning–based classification model to confirm MGUS and MM diagnoses of patients identified with *ICD-9* and *ICD-10* codes was a critical step to ensure study quality.^26,27,32^ This aspect of our analysis is important, as it has been shown that only 58% to 63% of patients with MM diagnoses based on *ICD-9* and *ICD-10* codes in the VHA system truly had MM after verification of disease using manual health record review or verifying whether MM-specific treatment was received.^13,43^ The NLP model–confirmed MGUS and MM diagnosis increases the accuracy of cohort inclusion and outcome ascertainment compared with other studies relying solely on administrative data. We excluded patients that progressed within 6 months of MGUS diagnosis to exclude patients who may have already had smoldering MM or undiagnosed MM.

### Limitations

This study has some limitations. There is a potential for lead-time bias, as individuals with BMI 25 or greater frequently present with comorbidities, like diabetes and hypertension, leading to more frequent clinic visits and laboratory checks and possibly earlier MGUS detection. Previous studies also observed that a higher comorbidity score and an elevated creatinine level had a lower risk of progression due to the artificially inflated time between MGUS and MM diagnoses.^13,44^ This bias cannot be reversed even if we control for potential confounders. Nonetheless, compared with using other databases, this bias would be smaller, as the VHA provides relatively equal health care access. The inclusion of only Black and White patients can limit the generalizability of this study; however, it is difficult to assess other racial groups due to low sample sizes, and race and ethnicity data were self-reported, which limited further investigation of the underpinnings of racial and ethnic disparities. Nonetheless, we were able to study a large population of Black patients, which contributes to many challenges in MM health disparities. Additionally, diet, lifestyle, smoking, and exercise levels can be confounders and interact with the association of obesity or weight gain with progression, as noted in a few studies.^41,45,46^ However, these factors are difficult to incorporate due to data unavailability and limitations that would likely significantly reduce our sample sizes. Along a similar line, determining whether the relationship between cumulative exposure of EBMI and MGUS progression is causal is out of the scope of this study. However, given the preponderance of data demonstrating the association of lifestyle and/or behavioral factors, environmental exposures, obesity, diabetes, and diabetes medication use with progression,^13,14,23,41,44,45,46,47,48,49,50^ our data further inform this evolving evidence. Our study is also unable to elucidate the cellular mechanisms driving the association between cumulative EBMI and progression of MGUS to MM. However, prior translational studies have identified mechanisms behind obesity and myelomagenesis.^16,17^ Moreover, our multivariable analyses did not include free light chain ratio and bone marrow plasma cell percentages as covariables since this was not uniformly obtained from 1999 to 2021 in patients with MGUS and would lead to lower sample size. Furthermore, our results may not be generalizable to the general US population, given the characteristics of the VHA population.^51,52,53^ For instance, a previous population-based study reported that the association between BMI and progression is stronger in females but our study may not accurately reflect this association, given the low number of females.^39^

## Conclusions

In this cohort study of US veterans, for patients with BMI of 18.5 to less than 25 at MGUS diagnosis, cumulative exposure to EBMI following MGUS diagnosis was associated with an increased risk of progression to MM. This suggests that these patients should avoid gaining weight and prolonged exposure to EBMI and could benefit from more stringent follow-up and targeted counseling or intervention aimed at maintaining a BMI within the reference range. Future research will help elucidate the relationship between healthy, safe weight loss and MGUS to MM progression risk.

## Notes

Supplement 1. **eFigure.** Area Under the Curve of BMI Trajectories **eTable 1.** List of Myeloma-Specific Treatments **eTable 2.** Multivariable Analysis of EBMI and MGUS to MM Progression Risk Stratified by BMI Category

Supplement 2.
Data Sharing Statement
