# Supplementary material for: Cumulative Excess Body Mass Index and MGUS Progression to Myeloma
Source: JAMA Netw Open. 2025 Feb 7;8(2):e2458585. doi: 10.1001/jamanetworkopen.2024.58585 (PMC11806393; doi:10.1001/jamanetworkopen.2024.58585)
Supplement: Supplement 1. — eFigure. Area Under the Curve of BMI Trajectories eTable 1. List of Myeloma-Specific Treatments eTable 2. Multivariable Analysis of EBMI and MGUS to MM Progression Risk Stratified by BMI Category [file jamanetwopen-e2458585-s001.pdf]

Supplemental Online Content

Liu L, Grandhi N, Wang M, et al. Cumulative excess body mass index and MGUS progression to myeloma. *JAMA Netw Open*. 2025;8(2):e2458585. doi:10.1001/jamanetworkopen.2024.58585

eFigure. Area Under the Curve of BMI Trajectories

eTable 1. List of Myeloma-Specific Treatments

eTable 2. Multivariable Analysis of EBMI and MGUS to MM Progression Risk Stratified by BMI Category

This supplemental material has been provided by the authors to give readers additional information about their work.

© 2025 Liu L et al. *JAMA Network Open*.

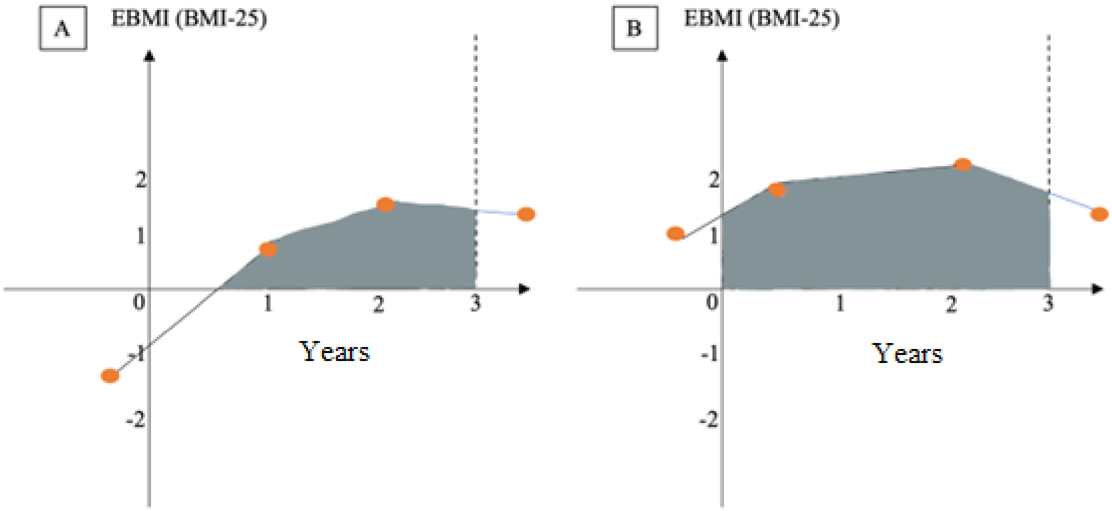

eFigure. Area Under the Curve of BMI Trajectory Sample Graph. A: For patients with BMI of 18.5 to less than 25 at MGUS diagnosis. B: For patients with BMI 25 or greater at MGUS diagnosis. Only positive AUCs were included in the analysis and negative AUCs were considered 0. For patients with only one BMI measurement during the observation period, the body weight was considered stable. Abbreviations: excess body mass index, EBMI; body mass index, BMI; monoclonal gammopathy of undetermined significance, MGUS.

© 2025 Liu L et al. *JAMA Network Open*.

eTable 1. List of myeloma-specific treatments:

abecma
belamaf
belantamab
belmaf
bendamustine
blenrep
bortezomib
carfilzomib
carvykti
cevostamab
ciltacabtagene
cilta-cel
cyclophosphamide
cytoxan
daratumumab
darzalex
elotuzumab
elranatamab
empliciti
evomela
farydak
idecabtagene
ide-cel
isatuximab

© 2025 Liu L et al. *JAMA Network Open*.

ixazomib
kyprolis
lenalidomide
melflufen
melphalan
ninlaro
panobinostat
pomalidomide
pomalyst
revlimid
sarclisa
selinexor
talquetamab
teclistamab
tecvayli
thalidomide
thalomid
velcade
venclexta
venetoclax
xpovio

© 2025 Liu L et al. *JAMA Network Open*.

eTable 2. Multivariable analysis of EBMI and MGUS to MM progression risk stratified by BMI category at diagnosis of MGUS.

AUC of EBMI, per unit increase	Baseline BMI			
	18.5 to less than 25	25 to less than 30	30 or greater	18.5 or less
aHR (95% CI)*	1.16 (1.01-1.34)	0.99 (0.90-1.08)	0.99 (0.97-1.00)	1.06 (0.86-1.31)

*Stratified by baseline BMI and adjusted for the following: age at MGUS diagnosis, sex (male, female), race (Black, White), MGUS type (Ig A, IgG, light-chain), M-protein level at MGUS diagnosis (<1.5 g/dL or ≥1.5g/dL), presence of diabetes mellitus, presence of anemia, presence of chronic kidney disease, and Charlson comorbidity index.

Abbreviations: excess body mass index, EBMI; body mass index, BMI; monoclonal gammopathy of undetermined significance, MGUS; multiple myeloma, MM, area under the curve, AUC; adjusted hazard ratio, aHR; confidence interval, CI.

© 2025 Liu L et al. *JAMA Network Open*.
