# Supplementary material for: Cumulative Excess Body Mass Index and MGUS Progression to Myeloma
Source: JAMA Netw Open. 2025 Feb 7;8(2):e2458585. doi: 10.1001/jamanetworkopen.2024.58585 (PMC11806393; doi:10.1001/jamanetworkopen.2024.58585)
Supplement: Supplement 2. — Data Sharing Statement [file jamanetwopen-e2458585-s002.pdf]

Data Sharing Statement

Liu. Cumulative Excess Body Mass Index and MGUS Progression to Myeloma. *JAMA Netw Open*. Published February 07, 2025. doi:10.1001/jamanetworkopen.2024.58585

Data
Data available: No

Additional Information
Explanation for why data not available: Data from the VHA cannot be shared publicly.
